# Supplementary material for: The interactome of intact mitochondria by cross-linking mass spectrometry provides evidence for coexisting respiratory supercomplexes
Source: Mol Cell Proteomics. 2017 Dec 8;17(2):216–32. doi: 10.1074/mcp.RA117.000470 (PMC5795388; doi:10.1074/mcp.RA117.000470)
Supplement: Supplemental Data [file supp_RA117.000470_133922_0_supp_23978_fzffwf.zip › spectra_annotation/mito_DR_spectra_annotation/360-1-1-1-7-1.pdf]

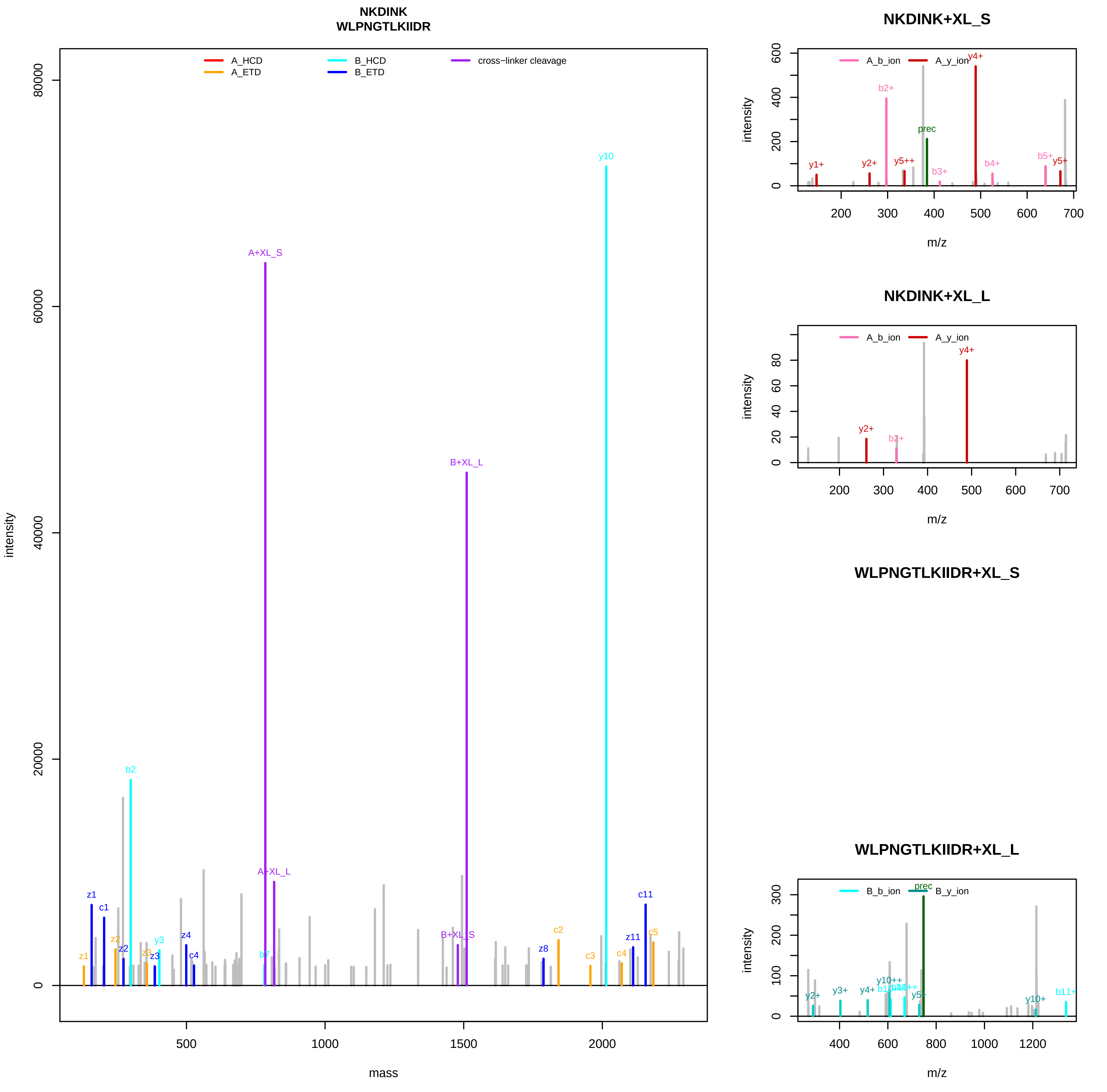
NKDINK
WLPNGTLKIIDR
A_HCD
A_ETD
B_HCD
B_ETD
cross−linker cleavage
intensity
mass
0
20000
40000
60000
80000
500
1000
1500
2000
y10
A+XL_S
B+XL_L
b2
A+XL_L
B+XL_S
z1
c1
z2
z3
y3
z4
c4
b7
z8
c2
c3
c4
z11
c11
c5
NKDINK+XL_S
A_b_ion
A_y_ion
y4+
b2+
prec
y1+
y2+
y5++
b3+
b4+
b5+
y5+
m/z
NKDINK+XL_L
y2+
b2+
WLPNGTLKIIDR+XL_S
WLPNGTLKIIDR+XL_L
B_b_ion
B_y_ion
y2+
y3+
y4+
y10++
y5+
y10+
b11+
